# Inhibition of Calpains Protects Mn-Induced Neurotransmitter release disorders in Synaptosomes from Mice: Involvement of SNARE Complex and Synaptic Vesicle Fusion

**DOI:** 10.1038/s41598-017-04017-9

**Published:** 2017-06-16

**Authors:** Can Wang, Bin Xu, Zhuo Ma, Chang Liu, Yu Deng, Wei Liu, Zhao-Fa Xu

**Affiliations:** Department of Environmental Health, School of Public Health, China Medical University, Shenyang, 110122 People’s Republic of China

## Abstract

Overexposure to manganese (Mn) could disrupt neurotransmitter release via influencing the formation of SNARE complex, but the underlying mechanisms are still unclear. A previous study demonstrated that SNAP-25 is one of substrate of calpains. The current study investigated whether calpains were involved in Mn-induced disorder of SNARE complex. After mice were treated with Mn for 24 days, Mn deposition increased significantly in basal nuclei in Mn-treated and calpeptin pre-treated groups. Behaviorally, less time spent in the center of the area and decreased average velocity significantly in an open field test after 24 days of Mn exposure. With the increase in MnCl_2_ dosage, intracellular Ca^2+^ increased significantly, but pretreatment with calpeptin caused a dose-dependent decrease in calpains activity. There were fragments of N-terminal of SNAP-25 protein appearance in Mn-treated groups, but it is decreased with pretreatment of calpeptin. FM1-43-labeled synaptic vesicles also provided evidence that the treatment with Mn resulted in increasing first and then decreasing, which was consistent with Glu release and the 80 kDa protein levels of SNARE complexes. In summary, Mn induced the disorder of neurotransmitter release through influencing the formation of SNARE complex via cleaving SNAP-25 by overactivation of calpains *in vivo*.

## Introduction

Manganese (Mn) is an essential trace element that is required for maintaining proper function and regulation of numerous biochemical and cellular reactions, it also functions as a cofactor for multiple enzymes^1^. Despite its essentiality, at excessive levels Mn is toxic to the central nervous system (CNS)^2^. Mn is not only a common industrial toxicant but an environmental pollutant. Occupational exposure to Mn has been recognized as a health hazard for miners, welders, ferroalloy workers, battery manufacturers and car mechanics^3^. Mn is also a component of an antiknock gasoline additive, known as methylcyclopentadienyl Mn tricarbonyl (MMT), and combustion results in release of Mn phosphates into the ambient air^4^. Mn is transported to the CNS either as a free ion or as a non-specific protein-bound species and accumulated in striatum, globus pallidus (GP) and the substantia nigra (SN)^5^. Overexposure to Mn from environmental sources can result in a condition known as manganism. Although oxidative stress, energy failure, and mitochondrial dysfunction have been actively investigated as neurotoxic mechanisms of Mn over the past two decades^6, 7^, emerging evidence indicates that disturbance of neurotransmitter release is also one of the important cellular and molecular correlates of neurodegenerative diseases resulting from chronic Mn exposure^8^.

A previous study demonstrated that manganese released into the synaptic cleft may influence synaptic neurotransmission, the levels of glutamate (Glu) and γ-aminobutyric acid (GABA) in the rat hippocampus, were dose-dependently decreased during treatment with manganese^9^. Several studies have established the propensity of Mn to disrupt Glu transporting systems, thus impairing components of the Gln/Glu-γ-aminobutyric acid cycle and leading to both a reduction in Glu uptake and elevation in extracellular Glu level^10^. Mechanisms of Mn disrupting neurotransmitter release are complicated and not firmly established. Therefore, understanding the exact molecular mechanisms of Mn disrupting neurotransmitter release may play a key role linking the complicated neurobehavioral deficits observe following Mn exposure.

SNAREs (soluble N-ethylmaleimidesensitive fusion protein attachment protein receptors) and associated proteins play critical roles in mediating neurotransmitter release^11^. The SNARE complex consists of three components. The vesicle-associated membrane protein 2 (VAMP-2/synaptobrevin) is a 116-amino acid protein anchored in the vesicle membrane by a single transmembrane domain. Syntaxin 1 is correspondingly anchored in the plasma membrane via a single transmembrane helix. The third component, SNAP-25, has lipid anchors in the plasma membrane^12^. SNARE proteins function in fusion by a cycle of assembly into complexes that fuel fusion, and disassembly of the complexes by NSF (N-ethylmaleimide sensitive factor) and SNAPs (soluble NSF-attachment proteins) that makes SNARE proteins available again for another round of fusion^13^. Our previous study had shown that Mn disturbed the expression and interaction of SNARE complex associated proteins and resulted in a decrease of protein expression of SNAP-25 in primary cultured neurons^14^, it’s mechanism needed to further study.

The mechanisms governing the proteolytic cleavage of SNAP-25 are not completely clarified, but a potential candidate protease is calpains. It belongs to a family of calcium-dependent, non-lysosomal, neutral, cysteine proteases, which cleave several cytosolic, membrane or cytoskeleton-associated proteins, seems to play an important role in synaptic plasticity^15^. Proteolysis by calpains is likely to change the integrity, localization, and/or activity of endogenous proteins, and results in either the activation or the inhibition of substrate functions. Recent data suggested that the component of the SNARE proteins SNAP-25 was substrates of members of the calpains family in neurons, and calpains may play an important role in the long-lasting regulation of synaptic vesicle fusion by suppressing neurotransmitter release, possibly through the proteolytic cleavage of SNAP-25^16^.

Although several studies have reported that Mn could disorder the formation of SNARE complex, little data exist on calpains involved in Mn-induced cleavage of SNAP-25, information that is critical for more fully evaluating the Mn-induced neurotoxicity. Therefore, in order to verify our speculation, calpeptin, an inhibitor of calpains, was being used in this study. We found that calpeptin could relieve Mn-induced SNARE complex formation disorders.

## Results

### Manganese concentrations and behavioral activity

**Figure 1:**
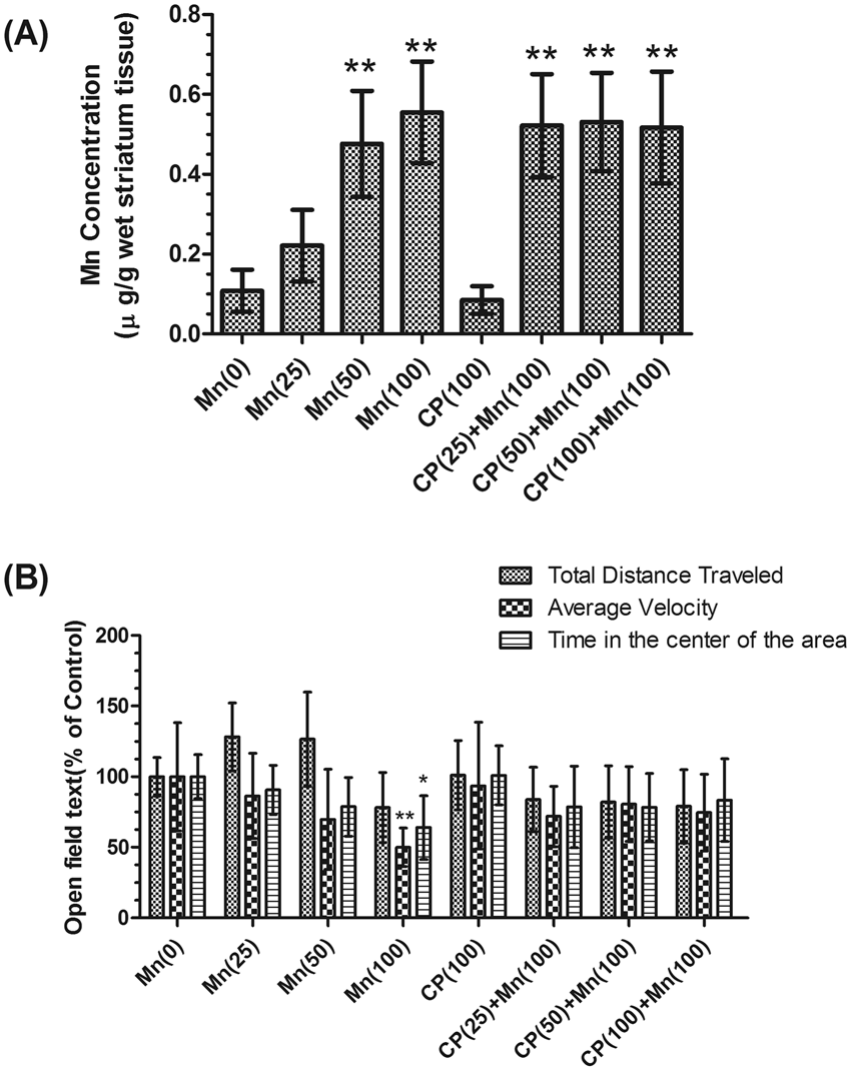
Manganese concentrations of mice basal nuclei and behavioral activity of mice on the 24th day after Mncl_2_-exposed. (**A**) Total Mn levels in basal nuclei of mice. Mice were intraperitoneally (i.p.) treated with physiological saline as control group and MnCl_2_ (25, 50, 100 μmol/kg) as Mncl_2_-exposed groups, subcutaneously (s.c.) pre-treated with 100 μg/kg calpeptin as calpeptin control group and calpeptin (25, 50, 100 μg/kg) accompany with 100  μmol/kg MnCl_2_ for 24 days, and 24 h after treatment was finished the basal nuclei were isolated. (**B**) Behavior in an open field test on the 24th day after Mncl_2_-exposed. Mice were placed in the center of the area for 2 min to acclimatization then recorded for 5 min. The total distance traveled, average velocity and time in the inner central zone were measured respectively. The values are expressed as means ± S.D., n = 6. *P < 0.05 and ^**^ *P* < 0.01significantly different form control group; ^#^ *P* < 0.05, significantly different form 100 μmol/kg MnCl_2_-treated group.

With the increase of MnCl_2_ dosage, there were significant increase in total Mn level in basal nuclei of 50 and 100 μmol/kg MnCl_2_-exposed mice (0.476 ± 0.133 and 0.555 ± 0.127 μg/g, respectively, *P* < 0.01) compared with control group, the same to the three calpeptin pre-treated groups (Fig. 1A). Behavioral measures were assessed at 8, 16 and 24 days during administration, we found that with increased Mn concentrations, mice were less active and spend less time in the center area, or take longer to initially explore the center area were considered to be displaying emotional alterations. Moreover, Mice in 100 μmol/kg MnCl_2_-exposed group exhibited a significant decrease in locomotor activity during exploration period of the open field test on the 24th day. Specifically, the average velocity was decreased by 53.3% (*P* < 0.01) and the time in the center area was decreased by 35.97% (*P* < 0.05) significantly. The total distance traveled have no significant change but it was decreased visibly. However, 25 and 50 μmol/kg MnCl_2_-exposed mice and all calpeptin pre-treated mice did not show any signs of abnormal behavioral activity at the end of the test (data not shown) (Fig. 1B). There was no significant difference between each group on the 8th day and the 16th day results. And There was no difference between female and male mice.

### Mn disrupted synapse structure and numbers of synaptic vesicles

**Figure 2:**
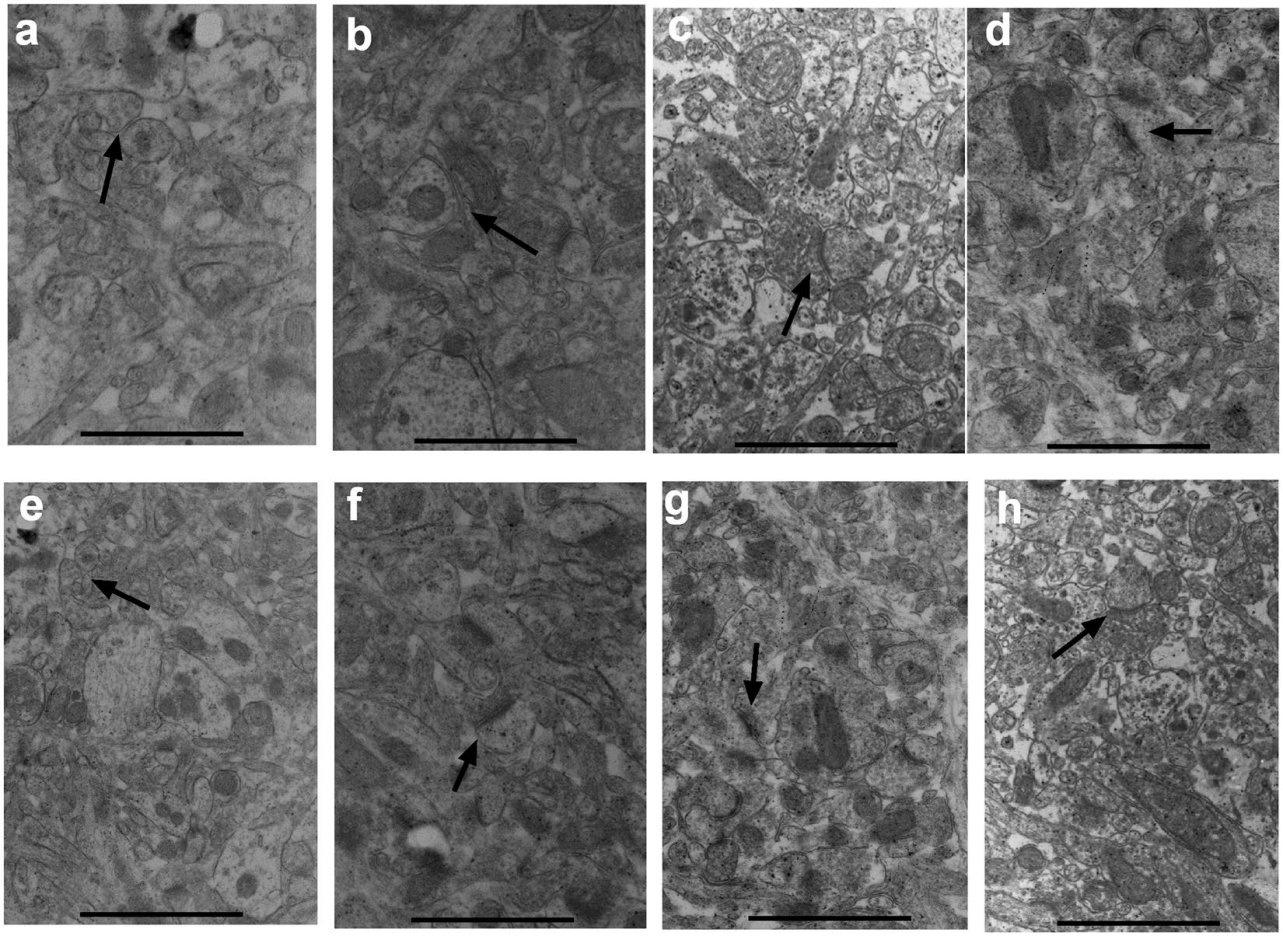
Transmission electron microscopy of synapse ultrastructure and numbers of synaptic vesicles, note the postsynaptic density (arrows). (**a**) synapse of control group, (**b**) 25 μmol/kg MnCl_2_-treated group, (**c**) 50 μmol/kg MnCl_2_-treated group, (**d**) 100 μmol/kg MnCl_2_-treated group, (**e**) calpeptin control group, (**f**) 25 μg/kg calpeptin pre-treated group, (**g**) 50 μg/kg calpeptin pre-treated group, (**h**) 100 μg/kg calpeptin pre-treated group. Magnification: ×10000; Scale bars represent 500 nm.

Pathological features of synapse ultrastructure and the numbers of synaptic vesicles were observed with Transmission Electron Microscopy (TEM). In the control group, normal synapse ultrastructure was observed in the basal nuclei: a clear synaptic cleft and less postsynaptic density (PSD), and certain numbers of synaptic vesicles in the presynaptic membrane (Fig. 2a). However, with the increase of administered-MnCl_2_ dosage, the number of synaptic vesicles decreased, the thickness of PSD in the synapses increased, and the synaptic cleft became blurred (Fig. 2b–d). In the calpeptin control group, the pathological features of synapses are similar to the control group (Fig. 2e). In the calpeptin pre-treated groups, the number of synaptic vesicles increased, the thickness of PSD in the synapses decreased, and the synaptic cleft became cleared with the increase of calpeptin pre-treat dosage (Fig. 2f–h).

### Effects of Mn and calpeptin pretreatment on intracellular Ca^2+^ and calpains activity in mice basal nuclei

**Figure 3:**
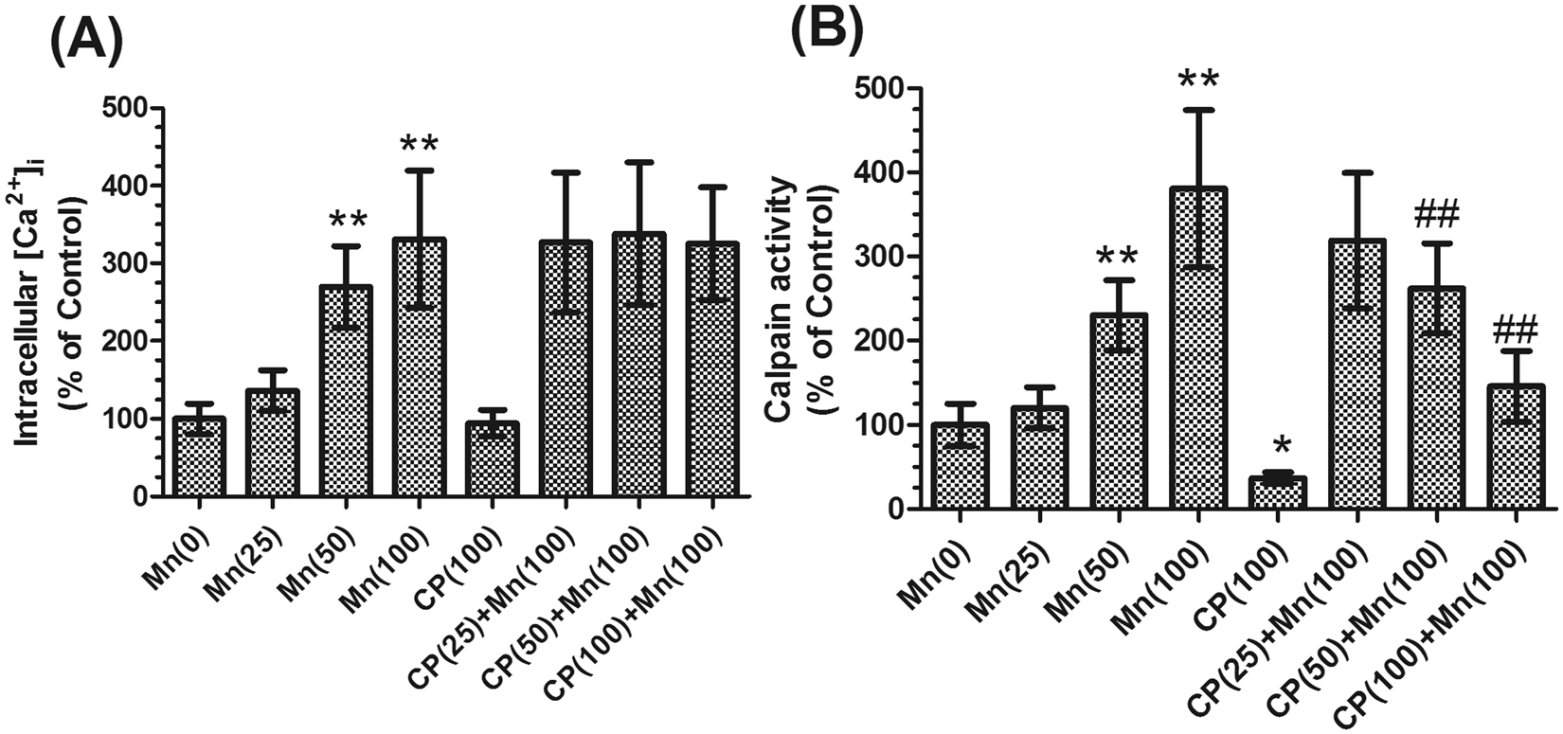
Effects of Mn and calpeptin pretreatment on intracellular Ca^2+^ and calpain activity in basal nuclei of mice. (**A**) [Ca^2+^]_i_ in the mice basal nuclei was calibrated from the measured fluorescence signals by the use of an F-4500 Fluorescence Spectrophotometer as described in the experimental section. (**B**) After brain was homogenized, calpain activity was measured using a spectrophotometer at 595 nm as described in the experimental section. Data are expressed as a percentage of controls, the values are expressed as means ± S.D., n = 6. ^*^ *P* < 0.05 and ^**^ *P* < 0.01, significantly different form control group; ^##^ *P* < 0.01, significantly different form 100 μmol/kg MnCl_2_-treated group.

Calpains are intracellular calcium-dependent proteases. To examine whether Mn affects calpains activity, we first measured the change of intracellular Ca^2+^ using the Ca^2+^ indicator Fura-2 AM. Treatment of mice with 50 and 100 μmol/kg MnCl_2_ resulted in a significant increase in [Ca^2+^]_i_ (2.69- and 3.31-fold of control respectively, *P* < 0.01, Fig. 3A). [Ca^2+^]_i_ was not significantly different in mice pretreated with calpeptin compared with 100 μmol/kg MnCl_2_-treated group, which were still higher than those in the absolute controls (Fig. 3A). Similar to the increase in [Ca^2+^]_i_, treatment with 50 and 100 μmol/kg MnCl_2_ also caused a significant increase in calpains activity compared with control (2.3- and 3.8-fold of control, respectively, *P* < 0.01, Fig. 3B). However, calpains activity decreased by 63.31% in mice treated with 100 μg/kg calpeptin alone compared with the controls (*P* < 0.05). Pretreatment with calpeptin caused a dose-dependent decrease in calpains activity, with the maximum decrease in the mice pretreated with 50 and 100 μg/kg calpeptin (31.19% and 61.66% respectively, *P* < 0.01, Fig. 3B), but calpains activity was still higher than those in the absolute controls. These data suggested that calpeptin could inhibit the activity of calpains. However, there was no effect on [Ca^2+^]_i_.

### Effects of Mn and calpeptin pretreatment on the release of neurotransmitters and synaptic vesicle fusion in synaptosomes

**Figure 4:**
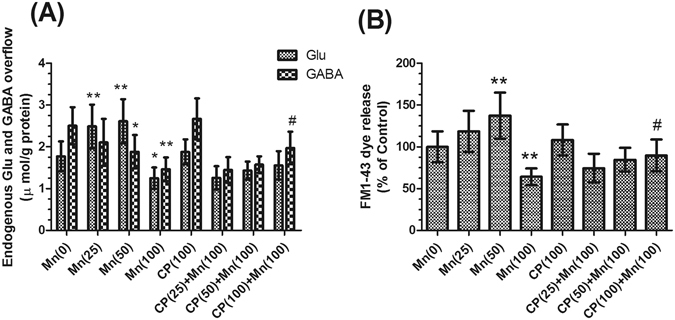
Calpeptin abated the Mn-induced decrease of neurotransmitters release. (**A**) Effects of MnCl_2_ and calpeptin pre-treatment on Glu and GABA release in basal nuclei by using HPLC. (**B**) Effects of MnCl_2_ and calpeptin pre-treatment on neurotransmitter release by using release of the fluorescent dye FM1-43. Data are expressed as a percentage of controls, the values are expressed as means ± S.D., n = 6. *P < 0.05 and **P < 0.01 significantly different form control group; ^#^ *P* < 0.05, significantly different form 100 μmol/kg MnCl_2_-treated group.

To examine the effect of manganese on the neurotransmitters release, endogenous Glu and GABA release in synaptosomes was measured by High Performance Liquid Chromatography (HPLC), with the increase of administered-MnCl_2_ dosage, Glu released increased in the 25 and 50 μmol/kg MnCl_2_-treated groups (1.40- and 1.47-fold of control group respectively, *P* < 0.01, Fig. 4A), and decreased by 29.62% in the 100 μmol/kg MnCl_2_-treated group (*P* < 0.05, Fig. 4A). GABA released was decreased sostenuto, it’s significantly in 50 and 100 μmol/kg MnCl_2_-treated groups (24.88% and 41.67% of control, *P* < 0.05 and *P* < 0.01, respectively, Fig. 4A). Pretreatment with 100 μmol/kg calpeptin caused an increase in GABA released (1.35-fold of 100 μmol/kg MnCl_2_-treated group, *P* < 0.05, Fig. 4A). To confirm that Mn affects neurotransmitter release, we examined the influence of Mn on KCl-triggered exocytosis by assaying the rate of destaining (loss of FM1-43 fluorescence intensity). It has shown that the fluorescence intensity of before and after KCl-evoked significantly increased in the 50 μmol/kg MnCl_2_-treated group (1.37-fold of control, *P* < 0.01, Fig. 4B), but decreased by 35.82% (*P* < 0.01) in the 100 μmol/kg MnCl_2_-treated group (Fig. 4B). Pretreatment with 100 μg/kg calpeptin caused a significant increase in FM1-43 dye release compared with 100 μmol/kg MnCl_2_-treated group (1.39-fold of 100 μmol/kg MnCl_2_-treated group, *P* < 0.05, Fig. 4B).

### Effects of Mn and calpeptin pretreatment on the expression of SNARE complex associated proteins

**Figure 5:**
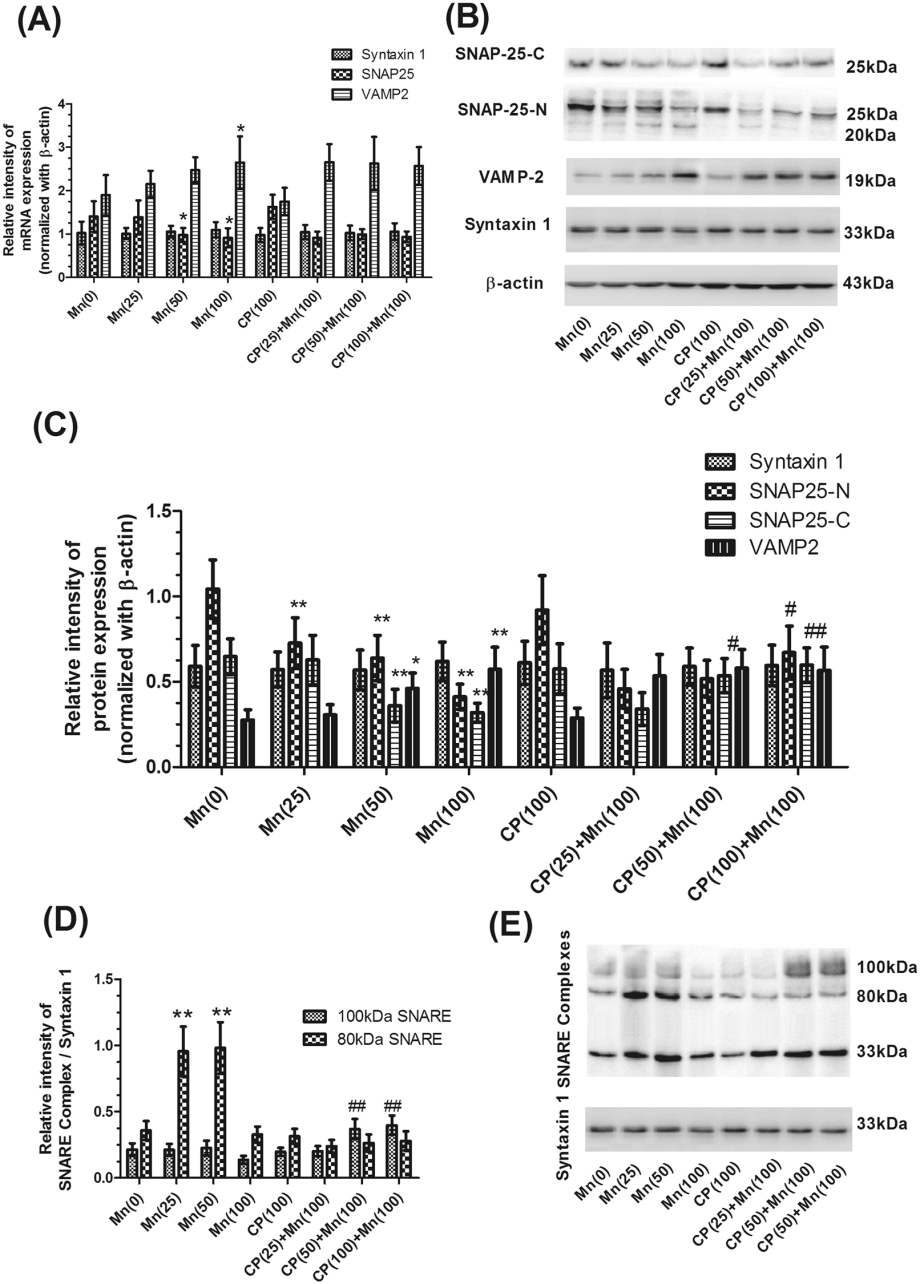
Calpeptin abated Mn disturbed the expression of SNARE complex associated proteins and the down-regulation of SNARE complex formation. (**A**) The mRNA expression levels were analyzed using a real-time RT-PCR assay to measure the expression of Syntaxin 1, SNAP-25 and VAMP-2 gene. Expression of Syntaxin 1, SNAP-25 and VAMP-2 gene were normalized withβ-actin gene. For relative quantification of the tested, we used the comparative CT method (△△CT). (**B**) The western blotting products of Syntaxin 1, N-terminal of SNAP-25, C-terminal of SNAP-25, VAMP-2 and β-actin. (**C**) Semi-quantitative analysis of the protein expressions of Syntaxin 1, N-terminal of SNAP-25, C-terminal of SNAP-25 and VAMP-2 were shown. Expression of Syntaxin 1, SNAP-25 and VAMP-2 protein were normalized with β-actin protein. (**D**) Semi-quantitative analysis of the protein expressions of SNARE complex protein was shown. Expression of SNARE complex protein was normalized with Syntaxin 1 protein. (**F**) The western blotting products of SNARE complex protein. Data are expressed as a percentage of controls, the values are expressed as means ± S.D., n = 6. *P < 0.05, **P < 0.01 significantly different form control group, ^#^ *P* < 0.05, ^##^ *P* < 0.01 significantly different form 100 μmol/kg MnCl_2_-treated group.

To investigate the molecular changes that could account for the neurotransmitter release disorders induced by Mn, we first measured the expression of the three presynaptic proteins (Syntaxin 1, SNAP-25 and VAMP-2) forming the SNARE core complex that mediates synaptic exocytosis. As shown in Fig. 5A, real-time PCR analysis showed that the expression of VAMP-2 mRNA significantly increased in a dosage-dependent manner, with the maximum increase in 100 μmol/kg MnCl_2_-treated group (1.40-fold of control, *P* < 0.05). Exposure of the mice to Mn also resulted in a marked decrease in the mRNA expression of SNAP-25 in 50 and 100 μmol/kg MnCl_2_-treated groups (30.44% and 35.12% respectively, *P* < 0.05). However, the mRNA expression of Syntaxin 1 did not significantly change in MnCl_2_-treated groups compared with control group, in addition, the mRNA expression of Syntaxin 1, SNAP25 and VAMP2 did not significantly change in calpeptin pre-treated groups compared with 100 μmol/kg MnCl_2_-treated group.

As shown in Fig. 5C, exposure of the mice to MnCl_2_ also resulted in a marked increase in the expression of VAMP-2 protein in 50 and 100 μmol/kg MnCl_2_-treated groups (1.67-, 2.08-fold of control, *P* < 0.05, *P* < 0.01, respectively). The analysis of SNAP-25 by using two different antibodies: a monoclonal antibody directed against the N-terminus of SNAP-25 (Synaptic Systems, N-terminal) and a polyclonal antibodies against the C-terminus of SNAP-25(Synaptic Systems, C-terminal). The expression of the N-terminus of SNAP-25 decreased significantly in 25, 50 and 100 μmol/kg MnCl_2_-treated group (30.36%, 38.73% and 50.50% respectively, *P* < 0.01). However, pretreatment with 100 μg/kg calpeptin caused an increase in the expression of N-terminus of SNAP-25 compared with 100 μmol/kg MnCl_2_-treated group (1.63-fold of 100 μmol/kg MnCl_2_-treated group, *P* < 0.05). The expression of the C-terminus of SNAP-25 have a marked decrease in 50 and 100 μmol/kg MnCl_2_-treated group (44.36% and 50.78% respectively, *P* < 0.01). However, pretreatment with 50 and 100 μmol/kg calpeptin caused an increase in the expression of C-terminus of SNAP-25 compared with 100 μmol/kg MnCl_2_-treated group (1.68- and 1.87-fold of 100 μmol/kg MnCl_2_-treated group, *P* < 0.05 and *P* < 0.01, respectively). However, the proteins expression of Syntaxin 1 did not have significantly change in MnCl_2_-treated groups as well as calpeptin pretreated groups.

### Effects of Mn and calpeptin pretreatment on the SNARE complex formation

To assess whether the number of SNARE core complexes was decreased in synaptic membranes of MnCl_2_-treated mice, we measured the amount of complexes, which were detected by loading not boiled synaptic membrane proteins on 4–20% gradient gels and developing Western blots with Syntaxin 1 antibody^17^. Two major syntaxin-1-containing complexes, migrating at 100 kDa and 80 kDa were detected (Fig. 5A). The 25 and 50 μmol/kg MnCl_2_-treated groups caused a significant increase in 80 kDa proteins levels of SNARE complexes versus controls (2.81- and 2.86-fold respectively, *P* < 0.01), and decreased by 15.18% (*P* < 0.05) in the 100 μmol/kg MnCl_2_-treated group. The 80 kDa protein in calpeptin pre-treated groups did not significant change, compared with 100 μmol/kg MnCl_2_-treated group (Fig. 5D). The 100 kDa protein levels of SNARE complexes did not significantly change in MnCl_2_-treated groups, compared with control group. The 50 and 100 μg/kg calpeptin pre-treated groups caused a significant increase in 100 kDa proteins versus 100 μmol/kg MnCl_2_-treated group (1.62- and 1.70-fold of 100 μmol/kg MnCl_2_-treated group, respectively, *P* < 0.01, Fig. 5D).

## Discussion

Our study has attempted to evaluate that Mn induced the disorder of neurotransmitter release through influencing the formation of SNARE complex via cleaving SNAP-25 by overactivation of calpains in basal nuclei, which is critical for evaluating the Mn-induced neurotoxicity.

In our mouse model of manganism, there were significant increase in Mn concentrations in the basal nuclei of both Mn-treated and calpeptin pre-treated mice compared with controls. To further confirm irreversible neurodegeneration and facilitate quantification following Mn intoxication, behavior was assessed by an open field test. In the present study, Mn-exposed mice were anxiety during the exploratory behavior in open field test. The locomotor activity during open field test in rodents is generally regarded as an indicator of emotional response to an unfamiliar environment, with an assumption that reduction in activity correlates with increased anxiety^18^. Given the results demonstrated in our study, it could be inferred that Mn exposure produced emotional alterations. Moreover, the decreased time spent in the center of the open field area indicates an increased anxiety level induced by Mn exposure. Mn-exposed mice also exhibited significant decrease in average velocity, this result suggested impairment in locomotion or emotional alterations. The increased anxiety in the face of increased depressive behavior exhibited by the Mn-exposed mice is likely related to the Mn-caused neurotransmitter release disorder^19^, as we discuss in more detail later.

Neurotransmitter release relies on the number of available synaptic vesicles and the release probability. Accordingly, the principal ultrastructural alterations produced by the neurotoxic effect of Mn in basal nuclei were the decrease in the number of synaptic vesicles and the increase in the thickness of PSD^20^. FM1-43 provides quantitative information about recycling pools of vesicles^21^. Our study provided evidence that the treatment with Mn resulted in synaptic vesicle fusion increasing in low dosage and decreasing in high dosage. Multiple studies have demonstrated the effect of Mn overexposure on neurotransmitter systems, like GABA and Glu^22^. Hence, we assessed the effect of Mn exposure on the GABA and Glu release, and found that Glu released was increased in low Mn dosage but decreased in high dosage, which was consistent with the quantity of synaptic vesicle fusion, and GABA released was decreased with Mn exposure in synaptosomes. These findings lend more support to the notion that the neurotransmitter release may be the major driving force for the Mn-induced behavioral alterations observed in this study.

Indeed, the SNARE complex and associated proteins play a critical role in vesicle docking, priming, fusion and synchronization of neurotransmitter release at presynaptic membranes and it was established that the SNARE complex corresponds to the minimal machinery for membrane fusion in eukaryotic cells, forming a stable complex that make the vesicles competent for fusion^23^. Therefore, a variation of SNARE complex accumulation in synaptic membranes is connected with disorder of neurotransmitter vesicle release. Syntaxin 1, SNAP-25 and VAMP-2 proteins are all SNARE proteins which exist in all types of synapses and play a central role in the release of neurotransmitters by synaptic vesicle exocytosis^11^. However, our study provided evidence that the treatment with Mn resulted in a dose-dependent decrease in the expression of SNAP-25 and increase in the expression of VAMP-2 but did not modify the mRNA and proteins expression of Syntaxin 1. The three proteins arrange as a tightly twisted, supercoiled, four-helical bundle with VAMP-2 anchoring in the vesicular membrane and SNAP-25 and Syntaxin 1 in the plasma membrane. The highly controlled interaction between vesicular and plasma membrane proteins is achieved by the formation of the ternary SNARE complex.

Calpains cleaves various types of presynaptic proteins: vesicular glutamate transporters, glutamatergic presynaptic sites^24^; GABAergic presynaptic sites such as glutamic acid decarboxylase, vesicular GABA transporter; plasma membrane GABA transporters^25^; and other important presynaptic proteins, such as synaptophysin and SNAP-25^26^. Numerous studies have shown presynaptic effects of calpains on glutamatergic and GABAergic synapses, targeting membrane-associated proteins as well as intracellular proteins. The resulting changes in the presynaptic proteome alter neurotransmitter release. These alterations also disturb the balance between excitatory and inhibitory neurotransmission in the brain^27^. Previous studies have found that the calpains cleavage site in SNAP-25 should be located at the C-terminal side^16^, thus proteins tested by C-terminal antibodies represent the full length of SNAP-25, and proteins tested by N-terminal antibodies are N-terminal fragments of SNAP-25. In the present study, treatment with Mn caused N-terminal fragments of SNAP-25 appeared and significant decrease in the number of SNAP-25. Strikingly, compared with 100 μmol/kg Mn-treated groups, there was a significant increase in the full length of SNAP-25 and N-terminal fragments were disappeared in 100 μM calpeptin pretreated mice, which could be explained by inhibited calpains activity.

Calpains are upregulated in a wide range of pathophysiological conditions characterized by dysregulation of neuronal calcium homeostasis, including stroke, epilepsy, traumatic brain injury and neurodegenerative disorders^28, 29^ and they are, indeed, recognized to be important mediators of neurotransmitter release disorder^30^. Small calcium changes falling into the physiological range can already result in significant variations of calpains activity^15^. Once they are activated by increased cytosolic Ca^2+^ load, calpains degrade a large number of cellular proteins, including SNAP-25 protein ultimately leading to neurotransmitter release disorder. ATP-driven plasma membrane calcium-pump (Ca^2+^-ATPase) is of crucial importance in maintaining a low resting intracellular Ca^2+^ concentration^31^. Previous studies have indicated Mn preferentially accumulates in mitochondria, where it disrupts oxidative phosphorylation and increases the generation of reactive oxygen species (ROS)^32^. Furthermore, it has been demonstrated that ROS overproduction might inhibit Ca^2+^-ATPases and this leads to altered regulation of Ca^2+^ levels^33^. Therefore, the inhibitory effect of Mn^2+^ on Ca^2+^-ATPases might be one of the reasons that causes a significant increase of [Ca^2+^]_i_ in neuron. Our previous studies also have found that overactivation of N-methyl-D-aspartate receptors (NMDARs), a phenomenon known as excitotoxicity, by Mn could result in an influx of extracellular Ca^2+^, which triggers a series of toxic events^34^. In addition to the inflow of extracellular calcium can lead to increased [Ca^2+^]_i_, endoplasmic reticulum(ER) stress can also lead to that. Our previous study have reported that Mn could induce ER stress^35^. Nearly 30% of proteins are not folded properly and misfolded proteins are retained in the ER, translocated to cytoplasm and degraded by proteasome (ER associated degradation). An abnormal protein burden in ER can lead to release of intracellular Ca^2+^ from ER, leading to mitochondrial Ca^2+^ uptake^36^. In this study, *in vivo* treatment of mice with Mn resulted in a significant increase in [Ca^2+^]_i_ and the activity of calpains. The results showed that calpains activity was excessively activated by Mn. It has been shown that the calpain-mediated SNAP-25 fragmentation correlates with a reduction of the SNARE function and inhibition of neurotransmitter release.

Calpeptin, an inhibitor of calpains, was used in this study. Therefore, our results demonstrated that calpeptin pretreatment significantly decreased in calpains activity, as well as partially increased SNARE complex protein formation. Moreover, there was none N-terminal of SNAP-25 in 100 μg/kg calpeptin pre-treated group. To confirm that calpeptin is non-toxic in low doses, we designed the present study to include a calpeptin alone group. The results demonstrated that there were no statistically significant differences in mice that were treated with calpeptin alone compared with controls. Based on these results, we concluded that low doses of calpeptin not only could inhibit the pathological consequences of calpains overactivation but also preserve cleavage of SNAP-25.

Taken together, the results of this study showed that Mn induced the disorder of neurotransmitter release through influencing the formation of SNARE complex via cleaving SNAP-25 by excessive activation of calpains *in vivo*. Moreover, inhibition of calpains could partially inhibit Mn-induced neurotransmitter release disorder. Our results give insight into the neurochemical alterations that take place in basal nuclei during elevated Mn exposure, and inhibition of calpains may represent a novel therapeutic target to ameliorate neuronal damage in manganism.

## Material and Methods

### Material

Manganese (II) chloride tetrahydrate (MnCl_2_·4H_2_O), Calpains, Calpeptin, SynaptoGreen^TM^C4 (FM1-43) and L-glutamine were purchased from Sigma (Saint Louis, MO, USA). PrimeScript^®^RT Enzyme Mix I and SYBR^®^Premix Ex Taq^TM^II kits were from TaKaRa Biotech. Co. Ltd. Mouse β-actin primary antibodies were purchased from Santa Cruz Biotechnology, Inc. (Santa Cruz, CA). Rabbit Syntaxin 1, VAMP-2 primary antibody were purchased from Abcam Ltd. (Hong Kong), mouse SNAP-25 N-terminal monoclonal and C-terminal polyclonal antibodies were purchased from Synaptic Systems (Germany). Horseradish peroxidase (HRP) conjugated anti-rabbit secondary antibody and HRP conjugated anti-mouse secondary antibody were purchased from Abcam. Additional chemicals of analytical grade were obtained from local chemical suppliers.

### Animal Procedures

A total of 24 male and 24 female adult Kunming mice weighing 25 ± 2 g were obtained from the Laboratory Animal Center of China Medicine University (SPF grade, Certificate No. SCXK 2013–0001). They were housed under conventional conditions at a room temperature of 21–24 °C, with a 12 h light: 12 h dark cycle and humidity of 30–40%. They were permitted free access to food and water. The animal experiment was carried out according to the National Institutes of Health Guidelines for the Care and Use of Laboratory Animals and approved by the local authorities. All efforts were made to minimize the number of animals used and their suffering. The mice were randomly divided into eight groups of six mice (three male and three female): control group, MnCl_2_-exposed groups (25, 50, 100 μmol/kg), calpeptin control group, and calpeptin pre-treated groups (25, 50, 100 μg/kg). The control group mice were intraperitoneally (i.p.) injected with physiological saline (group 1). MnCl_2_-exposed mice were respectively i.p. injected with 25, 50 and 100 μmol/kg MnCl_2_ in sterile deionized water (group 2, 3, 4). The calpeptin control group mice were subcutaneously (s.c.) injected with 100 μg/kg body weight calpeptin solution, 2 h before the i.p. administration with physiological saline (group 5). The calpeptin pre-treated mice were respectively s.c. injected with 25, 50, 100 μg/kg calpeptin solution, 2 h before the i.p. administration with 100 μmol MnCl_2_/kg bodyweight (group 6, 7, 8). The volume of injection was 2 ml/kg body weight, once every day, continuous injection for 24 days. For biochemical analysis, mice were killed by decapitation. The experimental protocols were approved by the Laboratory Animal Center of China Medicine University.

### Open field test

In order to assess possible effects of drug treatment on spontaneous locomotor activity, the animals were submitted to the open field paradigm as previously described^37^. Briefly, the open field area (50 × 50 × 50 cm) with a video camera attached to a computer was used to record animal behaviors, measured using EthoVision XT 11 software. 8, 16, 24 days after the administration, each mouse was placed individually into the center of the area and permitted free exploration. For analysis, the open field area was divided into two squares, the inner central zone (25 × 25 cm) and the outer periphery zone (25 cm from the walls). The mouse was positioned in the center of the area for 2 min to acclimatization then recorded for 5 min. The total distance traveled, average velocity and time in the inner central zone were measured respectively. Between each subject, the chambers were thoroughly cleaned and wiped down with 70% ethanol.

### Measurement of Mn concentration in basal nuclei

A weighed amount of tissue was wet-digested with 500 μl HNO_3_ (70% HNO_3_ for trace metal analysis). After partial evaporation, samples were cooled down, 500 μl H_2_O_2_ (36.5–38.0% for trace metal analysis) was added and the solution was totally evaporated. The precipitate was dissolved in 5 ml deionized water and analysis was performed by a HITACHI 180−80 atomic absorption spectrophotometer. Concentrations were measured using a standard calibration curve.

### Transmission Electron Microscopy

The basal nuclei specimens (1 mm³) were dissected from the basal neclei area 25 days after drug treatment. Briefly, the samples were placed in 2.5% glutaraldehyde and post-fixed with 1% osmium tetroxide. After graded ethyl alcohols, the cubes were embedded in Epon618. Thin sections laid on copper mesh were stained with heavy metals, uranyl acetate, and lead citrate for contrast. A Hitachi-H7650 transmission electron microscope (TEM; Hitachi, Japan) was used to observe the neural synapse ultrastructure.

### Measurement of intracellular free calcium

After preparation of the dissociated tissue, the [Ca^2+^]_i_ assay was performed by a method described previously^38^. Briefly, for fura-2 experiments, absolute values of [Ca^2+^]_i_ in the neurocyte were calibrated from the measured fluorescence signals using an F-4500 Fluorescence Spectrophotometer (Hitachi, Japan). Consequences of an individual experiment are reported. Similar data were obtained in six independent experiments. The calibration equation used was: [Ca^2+^]_i_ = K_d_ [(R-R_min_)/(R_max_-R)] × (S_f380_/S_b380_). [Ca^2+^]_i_ is the concentration (nM) of intracellular Ca^2+^; K_d_ is the dissociation constant of the dye; R is the ratio at excitation wavelengths 340/380 nm; R_min_ is the ratio at zero [Ca^2+^]_i_; and R_max_ is the ratio at saturated [Ca^2+^]_i_. The procedures for obtaining R_max_ and R_min_ caused damage to cells and were therefore performed at the end of the experiments. R_max_ was obtained first by adding Triton X-100 (0.2%), making the cell membrane permeable to Ca^2+^ and allowing the extracellular and intracellular Ca^2+^ to equilibrate. Next, R_min_ was obtained by adding the chelator EGTA [ethylene glycol bis (β-aminoethyl ether)-N, N, N′, N′-tetraacetic acid; 20 mM] to chelate all Ca^2+^ inside and outside the cells. The present experiments were carried out at pH 7.4 and a temperature of 37 °C. A Kd value of 224 nM was used. The results are expressed as a percentage of the controls.

### Measurement of calpains activity

Calpains activity was assayed as described by Buroker-Kilgore and Wang^39^. Briefly, after the brain tissues were homogenized in an extraction medium containing 5 mM β-mercaptoethanol, 0.1 mM EDTA, lysocephalin 5 mM, DL-Dithiothreitol 10 mM and 20 mM Tris-HCl at pH 8.6, tissue homogenate was centrifuged at 1000× g for 10 min to remove the protein precipitate. Samples were incubated with the calpains substrate casein, and after removal of an aliquot, Coomassie brilliant blue G-250 dye reagent was added to the aliquot and was quantified using a spectrophotometer at 595 nm. Calpains activity was calculated as the difference between samples with and without Ca^2+^. The results are expressed as a percentage of the controls.

### Preparation of synaptosomes from mice brain

Percoll-purified synaptosomes were prepared using the basal nuclei of mice, as described previously^40^. The mice were killed by decapitation, and the basal nuclei were rapidly removed at 4 °C. Synaptosomes were prepared by Percoll density-gradient centrifugation techniques. Briefly, the basal nuclei was isolated and homogenized in a medium that contained 320 mM sucrose, pH 7.4. The homogenate was centrifuged at 3000 g (5000 rpm in a JA 25.5 rotor; Beckman Coulter, Inc., USA) for 10 min at 4 °C, and the supernatant was centrifuged again at 14,500 g (11,000 rpm in a JA 25.5 rotor) for 12 min at 4 °C. The pellet was gently resuspended in 8 ml of 320 mM sucrose, pH 7.4. Two milliliters of this synaptosomal suspension was placed into 3 ml Percoll discontinuous gradients containing 320 mM sucrose, 1 mM EDTA, 0.25 mM DL-dithiothreitol, and 3, 10 and 23% Percoll, pH 7.4. The gradients were centrifuged at 32,500 g (16,500 rpm in a JA 20.5 rotor) for 7 min at 4 °C. Synaptosomes sedimenting between the 10 and the 23% Percoll bands were collected and diluted in a final volume of 30 ml of HEPES buffer medium (HBM) consisting of 140 mM NaCl, 5 mM KCl, 5 mM NaHCO_3_, 1 mM MgCl_2_.6H_2_O, 1.2 mM Na_2_HPO_4_, 10 mM glucose, and 10 mM HEPES (pH 7.4). Protein concentration was determined using the Bradford assay. Synaptosomes were centrifuged in the final wash to obtain synaptosomal pellets with 0.5 mg protein. The synaptosomal pellets were stored on ice and used within 4–6 h.

### FM1-43 fluorescence image analysis

Synaptic vesicle fusion with the plasma membrane was measured using release of the fluorescent dye FM1-43, as described previously^41^. In brief, synaptosomes (0.5 mg/ml) were incubated in HBM with 1.2 mM CaCl_2_ for 2 min at 37 °C in a stirred test tube. FM1-43 (100 μM) was added 1 min before stimulation with 30 mM KCl. After 3 min of stimulation to load FM1-43, synaptosomes were washed twice in HBM that contained 1.2 mM CaCl_2_ and 1 mg/ml BSA to remove non-internalized FM1-43. Synaptosomes were then resuspended in 2 ml of HBM (plus 1.2 mM Ca^2+^), and incubated in a stirred and thermostated cuvette maintained at 37 °C in a Perkin-Elmer LS-50B spectrofluorimeter. Release of accumulated FM1-43 was induced by the addition of 30 mM KCl, and measured as the decrease in fluorescence upon release of the dye into solution (excitation 488 nm, emission 540 nm). Data points were obtained at 2-s intervals, and data presented as the Ca^2+^-dependent decrease in FM1-43 fluorescence. Any drugs were added after the dye-loading procedure, and the synaptosomes were preincubated with osthole or imperatorin for 10 min before depolarization with KCl.

### Endogenous Glu and GABA release

Endogenous neurotransmitter release was measured as previously reported^42, 43^. Synaptosomes (about 100 μg of protein) were layered on microporous filters at the bottom of a set of parallel superfusion chambers maintained at 37 °C. A 30-min period of stimulation was applied with 15 mM KCl substituting for an equimolar concentration of NaCl. Fractions collected were analyzed for endogenous Glu and GABA content. Amino acid release was expressed as μmol/g of protein. Endogenous Glu and GABA were measured by High Performance Liquid Chromatography (HPLC) analysis after precolumn derivatization with o-phthalaldehyde and separation on a ZORBAX Eclipse XDB-C18 reverse-phase chromatographic column (250 × 4.6 mm, 5 μm; at 30 °C; Agilent Technologies, USA) coupled with fluorometric detection (excitation wavelength, 250 nm; emission wavelength, 410 nm). Buffers and the gradient program were as follows: solvent A, methanol; solvent B, 0.1 M sodium acetate (pH 5.8); gradient program, 20% A for 2 min from the initiation of the program; 47% A in 2 min; isocratic flow, 7 min; 53% A in 9 min; isocratic flow, 3 min; 100% A in 12 min; isocratic flow, 6 min; flow rate, 1.0 ml/min.

### Quantitative real-time PCR analysis

The mRNA expression levels were analyzed using a real-time reverse-transcription polymerase chain reaction assay. Total RNA was isolated using TRIzol reagent (TaKaRa Biotech. Co. Ltd., China). The first strand cDNA was synthesized from 1 μg of total RNA by reverse transcriptase using PrimeScript® RT Enzyme Mix I (TaKaRa Biotech. Co. Ltd., China) and oligo (dT) primers according to the manufacturer’s protocol. Real-time quantitative PCR (qPCR) was performed by SYBR® Premix Ex TaqTM II kit (TaKaRa Biotech. Co. Ltd., China) using an ABI 7500 Real-Time PCR System (Applied Biosystems, USA). Two microliters of template cDNA was added to the final volume of 20 μl of reaction mixture. Real-time PCR cycle parameters were 30 sec at 95 °C followed by 40 cycles with denatured at 95 °C for 5 sec, annealing at 60 °C for 34 sec and elongating at 72 °C for 20 sec. There are the sequences of the specific primer sets: Syntaxin 1’s Sense prime is 5′-ACCGCTTCATGGATGAGTTC-3′ and Anti-sense primer is 5′-GAGCTCCTCCAGTTCCTCCT-3′, SNAP-25′s Sense prime is 5′-CTGGCATCAGGACTTTGGTT-3′ and Anti-sense primer is 5′-ATTATTGCCCCAGGCTTTTT-3′, VAMP-2’s Sense prime is 5′-CTGCACCTCCTCCAAATCTT-3′ and Anti-sense primer is 5′-CTTGGCTGCACTTGTTTCAA-3′, β-actin’s Sense prime is 5′-GGAGATTACTGCCCTGGCTCCTA-3′ and Anti-sense primer is 5′-GGAGATTACTGCCCTGGCTCCTA-3^20^. Expressions of selected genes were normalized with the gene for β-actin, which was used as an internal housekeeping control. For relative quantification of the genestested, we used the comparative CT method (ΔΔCT). All the real-time PCR experiments were performed in quadruplicate, and data were expressed as the mean of at least four independent experiments.

### Western blotting

Samples of each fraction were analyzed by SDS–PAGE followed by Western blot. Tissue were rinsed with PBS and total protein was extracted from the neurons using RIPA buffer (10 mM Na_2_HPO_4_, pH 7.2, 150 mM NaCl, 1% sodium deoxicolate, 1% Nonidet P-40, 0.1% SDS) containing protease inhibitors (1 mM phenylmethylsulfonyl fluoride, 0.2 Mm 1, 10-phenanthroline, 10 μg/mL pepstatin A, 10 μg/mL leupeptin, 10 μg/mL aprotinin, and 10 mM benzamidine). Protein concentrations were determined with the BCA reagent from Pierce. Equal amounts of protein (20 μg) were separated by 10% polyacrylamide gel electrophoresis and transferred to polyvinylidene difluoride (PVDF) membranes (Millipore, Ternicula, CA). PVDF membranes blocked overnight at 4 °C in TBST containing 5% bovine serum albumin fraction V. Following which, the membranes were rinsed briefly in TBST and incubated with primary antibody in TBST for a night at 4 °C. Single components of SNARE were detected by incubated with Syntaxin 1 (1:200), VAMP-2 (1:200) and β-actin (1:200). SNAP-25 cleavage products were detected by incubating filters in anti-SNAP-25 N-terminal monoclonal (directed against the epitope containing amino acid residues 1–20) and C-terminal polyclonal antibodies (directed against AAs 192–206) (1:1000; Synaptic Systems, Germany). Specific protein expression was then detected by incubating the washed membranes with HRP conjugated secondary antibodies (1:2000). Protein bands were visualized by using the ECL Western blotting chemiluminescent detection reagents and autoradiography. The intensity of the bands was evaluated semi-quantitatively by densitometry using image analyzing software (FluorChem v2.0). The changes of intensity of Syntaxin 1, VAMP-2, anti-SNAP-25 N-terminal monoclonal and C-terminal polyclonal antibodies proteins after MnCl_2_ treatment were normalized using the intensity obtained in the internal control bands (β-actin). Results of an individual experiment that reflects similar data obtained on at least three separate occasions.

### SNARE complex analysis

For detection of SDS-resistant SNARE complexes, Western blotting was performed on samples of electrophoresed protein extraction (non-boiled before gel loading)^17^, incubating PVDF membranes containing blotted proteins with rabbit polyclonal antibodies for Syntaxin 1 (1:1000). The membranes were incubated with anti-rabbit secondary antibody (1:2000), and immunoreactive bands visualized by chemiluminescent detection reagents (Pierce). All bands were normalized with Syntaxin 1 monomer level (normal Western blotting) in the same sample.

### Statistical analysis

Statistical analyses were performed using SPSS 18.0 and the results were expressed as the mean ± S.D. Differences between the means were determined by one-way ANOVA followed by a Student–Newman–Keuls test for multiple comparisons. The difference at either *P* < 0.05 or *P* < 0.01 was considered statistically significant.
